# Investigating the Role of Hippocampal BDNF in Anxiety Vulnerability Using Classical Eyeblink Conditioning

**DOI:** 10.3389/fpsyt.2015.00106

**Published:** 2015-07-24

**Authors:** Kellie L. Janke, Tara P. Cominski, Eldo V. Kuzhikandathil, Richard J. Servatius, Kevin C. H. Pang

**Affiliations:** ^1^Research Service, Neurobehavioral Research Laboratory, VA New Jersey Heath Care System, East Orange, NJ, USA; ^2^Graduate School of Biomedical Sciences, New Jersey Medical School, Rutgers Biomedical and Health Sciences, Newark, NJ, USA; ^3^Veterans Biomedical Research Institute, East Orange, NJ, USA; ^4^Department of Pharmacology, Physiology and Neuroscience, New Jersey Medical School, Rutgers Biomedical and Health Sciences, Newark, NJ, USA; ^5^Syracuse VA Medical Center, Syracuse, NY, USA

**Keywords:** hippocampus, dentate gyrus, TrkB, Arc, Wistar-Kyoto rat

## Abstract

Dysregulation of brain-derived neurotrophic factor (BDNF), behavioral inhibition temperament (BI), and small hippocampal volume have been linked to anxiety disorders. Individuals with BI show facilitated acquisition of the classically conditioned eyeblink response (CCER) as compared to non-BI individuals, and a similar pattern is seen in an animal model of BI, the Wistar-Kyoto (WKY) rat. The present study examined the role of hippocampal BDNF in the facilitated delay CCER of WKY rats. Consistent with earlier work, acquisition was facilitated in WKY rats compared to the Sprague Dawley (SD) rats. Facilitated acquisition was associated with increased BDNF, TrkB, and Arc mRNA in the dentate gyrus of SD rats, but learning-induced increases in BDNF and Arc mRNA were significantly smaller in WKY rats. To determine whether reduced hippocampal BDNF in WKY rats was a contributing factor for their facilitated CCER, BDNF or saline infusions were given bilaterally into the dentate gyrus region 1 h prior to training. BDNF infusion did not alter the acquisition of SD rats, but significantly dampened the acquisition of CCER in the WKY rats, such that acquisition was similar to SD rats. Together, these results suggest that inherent differences in the BDNF system play a critical role in the facilitated associative learning exhibited by WKY rats, and potentially individuals with BI. Facilitated associative learning may represent a vulnerability factor in the development of anxiety disorders.

## Introduction

Anxiety is the most commonly treated and prescribed for psychiatric condition in today’s society. Determining who is susceptible to developing anxiety disorders and how these vulnerabilities impact treatment efficacy is currently an active area of research. Individual differences play a crucial role in whether a person develops an anxiety disorder or not. Epidemiologic studies indicate that exposure to early childhood trauma and chronic stress increases one’s risk to developing anxiety disorders, whereas a behaviorally inhibited temperament, a small hippocampal volume, and more recently, dysfunction of hippocampal brain-derived neurotrophic factor (BDNF) are associated with inherent vulnerabilities. While various risk or vulnerability factors have been identified, the mechanisms by which they confer vulnerability are still unknown (1, 2).

Brain-derived neurotrophic factor is a neurotrophin that influences cell growth, cell differentiation, and synaptic modification (3, 4) and is highly expressed in the developing and adult hippocampus (5–8). Recently, a single nucleotide polymorphism (SNP) of the coding region of the BDNF gene (Val66Met) has been identified as a risk factor for anxiety disorders, including post-traumatic stress disorder (PTSD) (9, 10). The genetic variation resulting in a substitution of a valine to a methionine at codon 66 restricts intra-cellular trafficking and activity-dependent release of hippocampal BDNF. Individuals with the met allele have reduced hippocampal volume (11–14), deficits in hippocampal-dependent memory (15, 16), and altered responses to emotional stimuli (17, 18). Given that BDNF is released in an activity-dependent manner, BDNF may be a key factor in experience-dependent vulnerability to psychiatric disorders (19).

The link between an abnormal BDNF system and anxiety vulnerability may be through the hippocampus. A small hippocampal volume and impaired hippocampal-dependent learning are likely pre-existing conditions in those that develop PTSD, suggesting that a dysfunctional hippocampus is a vulnerability factor for PTSD (2, 20). PTSD patients with the Val66Met SNP were less responsive to cognitive behavioral therapy than those without the SNP (21), implicating an involvement of BDNF in extinction learning. In humans, abnormally low levels of BDNF are associated with a smaller hippocampal volume (22) and mood disorders including obsessive-compulsive disorder (23), and depression (24). The link between low levels of hippocampal BDNF and mood disorders has been dubbed the neurotrophin hypothesis, whereby enhancement in BDNF signaling is observed in the hippocampus after administration of antidepressants (25, 26). These results suggest an association between dysfunction of the BDNF system, small hippocampal volume, hippocampal learning impairment, and risk to develop mood disorders in humans.

Similar to humans, BDNF is important for normal function of the hippocampus in animals. A low amount of BDNF is associated with a smaller hippocampal volume (22). BDNF is important for adult neurogenesis in the dentate gyrus (27), and reduced BDNF impairs spatial memory and extinction of fear memories (28). Anxiety-related behaviors are also enhanced in the transgenic mouse reproducing the Val66Met SNP (Met66 allele) of humans (9, 29). These mice have smaller hippocampi, reduced activity-dependent secretion of BDNF, dendritic shrinkage in the DG, and impaired extinction of fear conditioning compared to wild-type mice. The Val66Met polymorphism has also been linked to reductions in NMDA transmission, and resistance to selective serotonin reuptake inhibitor (SSRI)-induced LTP and neurogenesis in the dentate gyrus (30, 31). Thus, low levels of BDNF protein or impaired BDNF release via a Val66Met SNP results in a smaller hippocampus, abnormal fear extinction, anxiety-related behaviors, and reduced efficacy of antidepressants.

Behavioral inhibition is a temperament characterized by withdrawal from and avoidance of novel social and non-social interactions (32) and is a vulnerability factor for developing anxiety disorders (33–35). The neurobiology of inhibited temperament has been heavily linked to alterations in amygdala, prefrontal cortex, and basal ganglia (36). Although less well-studied with respect to inhibited temperament, the hippocampus also demonstrates altered function in individuals with inhibited temperament (36). In particular, the interaction of the risk factor of childhood maltreatment and the inherent vulnerability of inhibited temperament was associated with increased activation of the hippocampus to novel faces with the strongest correlation in individuals who developed an anxiety disorder (37). Importantly, the activity in the amygdala to novel faces did not correlate to childhood maltreatment, suggesting the amygdala and hippocampus may contribute differently to inhibited temperament.

Reflective of altered hippocampal function in behavioral inhibition is the facilitation of non-hippocampal-dependent associative learning in individuals with inhibited temperament. The delay paradigm of classical conditioning of the eyeblink response (CCER) does not require the hippocampus (38), in contrast to the trace paradigm of CCER. In fact, hippocampal damage can facilitate acquisition of delay CCER (39), whereas similar damage impairs acquisition of trace CCER (40). Support that inhibited temperament is associated with hippocampal dysfunction is the finding that individuals scoring high on behavioral inhibition scales acquire delay classical conditioning faster than non-inhibited individuals (41–44). Similarly, the Wistar-Kyoto (WKY) rat, an animal model of behavioral inhibition, demonstrated facilitated acquisition of delay CCER (45). Thus, behaviorally inhibited temperament is associated with facilitated associative learning that may underlie anxiety vulnerability (46).

The WKY rat demonstrates inhibited temperament as evidenced by reduced exploration in the open-field test (47, 48) and freezing behavior in response to novel social and non-social stress (48, 49). Additionally, WKY rats are hyper-sensitive to stress (50–52) and acquire active avoidance more rapidly, to a greater extent, and more persistently than Sprague Dawley (SD) rats (53, 54). Avoidance is a common feature of all anxiety disorders, and greater persistent avoidant responding is reminiscent of individuals with anxiety disorders (55). The WKY rat has a smaller hippocampal volume compared to the non-inhibited rat strains (56), is impaired in hippocampal-dependent learning tasks (49, 57), and behaves similarly to rats with hippocampal damage (56, 58). The BDNF system may be abnormal in the WKY rat; serum BDNF levels in WKY, but not SD, rats decreased following stress (59), and SSRIs are less effective in WKY rats compared to SD rats in the Porsolt Swim test (60), similar to mice with low levels of BDNF or Val66Met SNP.

In summary, an impaired BDNF system is a vulnerability factor for anxiety disorders and affects normal hippocampal function. Inhibited temperament is also a vulnerability factor for anxiety disorders and is associated with facilitated acquisition of delay CCER in humans and animals. The present study was conducted to determine whether an impaired hippocampal BDNF system underlies facilitated CCER that is associated with inhibited temperament and anxiety vulnerability.

## Materials and Methods

Subjects were male SD and WKY rats obtained from Charles River, Kingston, NY, USA. They were approximately 3 months in age at the time of testing and maintained on a 12-h light/dark cycle with onset of light at 0700 h. All animals were tested during the light phase. Rats were housed individually in standard cages (16.5 in × 8.5 in × 8 in) with ad lib access to food and water and were acclimated upon arrival for at least 5 days prior to experimentation. All experiments were carried out in accordance with the Institutional Animal Care and Use Committee of the East Orange, New Jersey Health Care System, Veterans Affairs Medical Center.

### Surgery

Sprague Dawley and WKY rats were anesthetized with Nembutal (50 mg/kg i.p.), and supplemented as necessary. Guide cannulas (26 g, Plastics One, Roanoke, VA, USA) were implanted bilaterally (4 mm posterior and 2.5 mm lateral from bregma, and −3.1 mm ventral from brain surface) directed at the dentate gyrus region of the hippocampus. Each guide cannula was fixed to skull screws (stainless steel) using dental acrylic cement. A stylet was inserted into the guide cannula to keep the cannula patent.

Electrodes were implanted into the periorbital muscles for eyeblink conditioning. Four Teflon-coated, stainless steel wires (75 μm diameter, AM Systems) had the insulation stripped from one end that was inserted into the muscle. The other end of the wire was inserted into a plastic connector (Cannon Centi-loc, ITT Cannon, Santa Ana, CA) that was glued to three to four skull screws using dental acrylic. Two wires were used to record electromyography (EMG) and the other two wires delivered electrical stimulation.

Following the surgical procedure, sutures were used as needed and rats were post-operatively treated with flunixin meglumine (2.5 mg/kg, s.c.) for 2 days. Rats were allowed at least 4 days to recover from surgery.

### Classical conditioning of the eyeblink response

Eyeblink conditioning was conducted in a sound-attenuated chamber (27 cm × 29 cm × 43 cm) with a viewing window (Med Associates, St. Albans, VT, USA). The EMG signals were recorded from electrodes that were connected to a differential AC amplifier through a cable attached to the plastic connector on the rat’s head. EMG signals were filtered (300–500 Hz) and amplified (10,000X, A-M Systems Model 1700, Everett, WA, USA). Electrical stimulation of the periorbital muscles was delivered by a stimulus isolation unit (Coulbourn Instruments, Whitehall, PA, USA). A computer equipped with an A/D board and LabView software (National Instruments, Austin, TX, USA) controlled stimuli presentation and recording of EMG signals digitized at a sampling rate of 1000 Hz. One day prior to conditioning, freely moving rats were habituated to the apparatus for 30 min. During habituation, EMG signal quality was determined. Rats were conditioned for 1 or 2 days following habituation.

Rats were conditioned using a delay conditioning paradigm. Rats received 100 conditional stimulus (CS)-unconditional stimulus (US) paired trials per day. An auditory stimulus (500 ms, 82 dB white noise, 10 ms rise/fall) served as the CS. Electrical stimulation of the periorbital muscles (10 V, 10 ms) served as the US. CS and US co-terminated. The inter-trial interval (ITI) ranged from 15 to 35 s with an average of 25 s.

Electromyography was analyzed to determine the occurrence of eyeblinks using a custom designed script in S-Plus (version 6.1, Insightful Corporation, Seattle, WA, USA). For each trial, the 250 ms prior to the presentation of the CS was used as a baseline for each trial. An eyeblink, conditioned response (CR), was designated when the EMG activity exceeded a threshold amplitude following the CS onset and prior to the US onset. Threshold amplitude was equal to the mean amplitude of the baseline plus four standard deviations of the baseline activity. Any response recorded during the first 30 ms of the CS onset (250–280 ms) was not counted as a CR, as this time frame typically indicates an orienting response and represents less than 10% of eyeblinks. To evaluate the rate of acquisition, trials were grouped into five blocks of 20 trials per day. Analysis of variance (ANOVA) with repeated measures was used to analyze CR.

### BDNF administration

For animals receiving infusions prior to eyeblink conditioning, an infusion cannula (33 g, Plastics One, Roanoke, VA, USA) attached to a Hamilton syringe via polyethylene tubing (PE 50, Becton Dickinson, Sparks, MD, USA) was inserted into the guide cannula. Sterile saline (0.5 μl) or rhBDNF (0.5 μg/0.5 μl; R&D Systems, Minneapolis, MN) was administered (0.1 μl/min) into the dentate gyrus region of the hippocampus. After drug administration, the infusion cannula was allowed to remain in place for 5 min, and then removed and replaced with a stylet. Infusions were given approximately 45 min (40–50 min range) prior to the start of the eyeblink conditioning session. Saline or BDNF was infused prior sessions 1 and 2 of conditioning.

### Tissue extraction

Animals for RT-PCR analysis were sacrificed and the hippocampus was extracted approximately 1 h after Day 1 of eyeblink conditioning. Because BDNF levels fluctuate throughout the day, tissue collection was confined to 3 h after the onset of the light cycle, approximately between 10:00 a.m. and 1:00 p.m. After decapitation and rapid removal of the brain, CA1, CA3, and dentate gyrus regions of both hippocampi were dissected rapidly on ice, placed in microcentrifuge tubes, and stored in dry ice. Net wet tissue weight of the tissue was recorded. Samples were stored at −80°C pending analysis.

### RT-PCR

mRNA for BDNF, TrkB (high affinity BDNF receptor), and the immediate early gene Arc (activity-regulated cytoskeleton-associated protein) was measured using RT-PCR. Total RNA was isolated from the dentate gyrus by submerging in Trizol reagent and adding Zirconium disruption beads (Thomas Scientific, Swedesboro, NJ, USA). Supernatant was further processed and DNase treated as per manufacturer’s instructions (Direct-zol RNA mini-prep, Zymo Research, Irvine, CA, USA). The RNA concentration was quantified using the NanoDrop Spectrophotometer (NanoDrop, Wilmington, DE, USA). Total RNA was reverse transcribed by first denaturing 1 μg sample and 1 μl of 300 ng/μL RT primer at 65°C for 5 min and then chilling on ice. Next, 6 μl of 5× Superscript Buffer, 1.5 μl 0.1M DTT, 1.5 μl 10 mM dNTPs, 1 μl Superase In, and 1 μl Superscript III (Life Technologies, Invitrogen, Carlsbad, CA, USA) were added to the samples and incubated at 25°C for 10 min, followed by 45°C for 2 h. The RT reaction was terminated by heating at 70°C for 15 min and the cDNA stored at −20°C. RT-PCR was performed using Roche Lightcycler^®^ containing 3 μl of cDNA, 10 μl Taqman Universal PCR master mix, 1 μl of Taqman probe (Bdnf Taqman Probe, Rn02531967; Ntrk2 Taqman Probe, Rn01441749_ml; Arc Taqman Probe, Rn00571208_gl; 18S Taqman Probe, hs99999901_s1; Applied Biosystems, Grand Island, NY, USA), 1 μl of Bovine Serum Albumin (2.5 mg/mL; BioFire, Salt Lake City, UT, USA), and 5 μl dH_2_0.

The cycle threshold (CT) value was determined for each probe. Data for each target gene were assayed in duplicate and averaged, target values were normalized to the mean of the housekeeping gene 18S ribosomal RNA, which showed the lowest amount of variability across strain and treatment. Fold differences between samples for each gene product were calculated as follows:

2^(Sample with highest CT value for target gene-individualsample’s CT value for target gene)2^(Sample with highest CT value for 18S rRNA-individualsample’s CT value for 18S rRNA)

### Statistical analysis

Statistical analyses were conducted using Statistical Package for the Social Sciences (SPSS for Windows, Version 16, SPSS, Inc., Chicago, IL, USA). All results were considered significant at α = 0.05. Behavioral data for mRNA analysis were evaluated with a mixed design ANOVA for CR probability with blocks as a within-subject factor and strain as a between-subject factor. Average CR probability was calculated for blocks consisting of 20 trials, resulting in five blocks per session. Behavioral data for BDNF administration had a similar experimental design, but with the addition of treatment as a between subjects factor. mRNA data were analyzed using an ANOVA with strain and conditioning as between subjects factors. Separate analyses were conducted for BDNF, TrkB, and Arc mRNA in each hippocampal subregion. Only significant (*p* < 0.05) and trending (*p* < 0.1) results are reported.

## Results

### Learning-induced changes in hippocampal BDNF, TrkB, and Arc mRNA

#### Behavior

**Figure 1:**
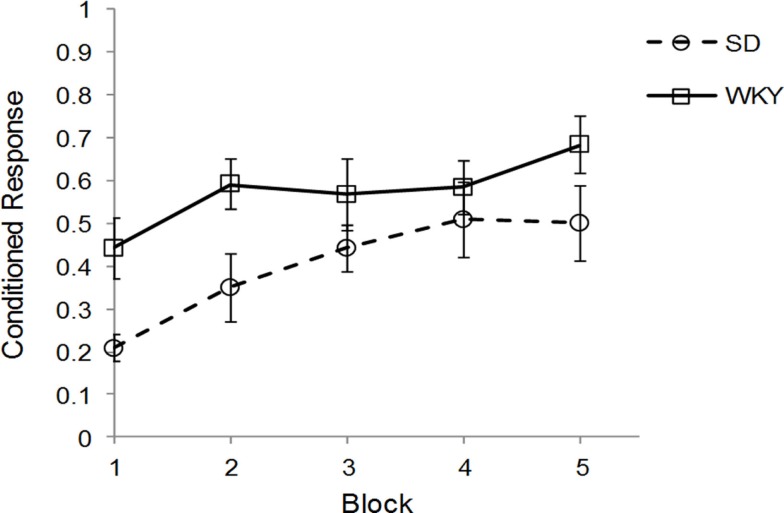
**Strain differences in classical eyeblink conditioning**. Wistar-Kyoto (WKY) and Sprague Dawley (SD) rats were trained in delayed classical conditioning of the eyeblink response. A session consisted of five blocks of 20 trials. WKY rats acquired eyeblink conditioning significantly faster and to a greater extent than SD rats, as demonstrated by higher levels of conditioned responses.

Sprague Dawley (*n* = 7) and WKY (*n* = 8) rats were trained in one session of delay classical conditioning of the eyeblink response followed by sacrifice for assessment of hippocampal BDNF, TrkB, and Arc mRNA. Due to problems with EMG recording, 1 SD and 2 WKY rats could not be evaluated for behavior; these rats showed clear eyeblink to periorbital electrical stimulation US and should demonstrate classical conditioning similar to other rats. Therefore, all rats were included in the mRNA analysis. Acquisition of classical conditioning was significantly faster and performed to a greater degree in WKY rats compared to SD rats, main effect of strain [*F*(1, 10) = 5.02, *p* < 0.05] (Figure 1), replicating previous results (45). Overall, general learning was demonstrated by a main effect of block [*F*(4, 40) = 8.38, *p* < 0.001]. No interaction between block and strain was observed. Ninety to one hundred and twenty minutes following the conditioning sessions, rats were sacrificed and the hippocampus removed, subdivided, and stored for subsequent analysis by qRT-PCR.

#### Brain-Derived Neurotrophic Factor

**Figure 2:**
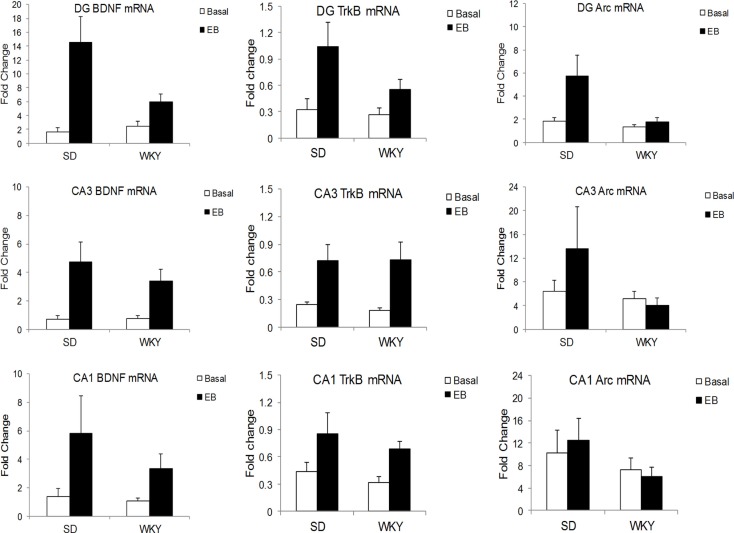
**Learning-induced increases in BDNF, TrkB, and Arc mRNA differed between SD and WKY rats**. Following a single session of classical conditioning of the eyeblink response (CCER), the hippocampal subregions were dissected, and BDNF (left), TrkB (middle), and Arc (right) mRNA was assessed in the dentate gyrus (upper), CA3 (middle), and CA1 (lower) subregions of the hippocampus. BDNF mRNA was significantly increased following acquisition of CCER in the dentate gyrus and CA3. In CA1, the main effect of learning did not reach significance (*p* = 0.074). Moreover, learning-induced changes in the dentate gyrus of WKY rats were significantly smaller than that in SD rats. By contrast, learning-induced changes of BDNF mRNA in CA3 were similar between strains. Learning caused increases of TrkB mRNA in all hippocampal subregions and increases were similar between SD and WKY rats. Finally, acquisition of CCER increased Arc mRNA only in the dentate gyrus, but not CA3 or CA1. The changes in Arc mRNA in the dentate gyrus were significantly smaller in the WKY rat compared to SD rat.

In the DG, learning increased BDNF mRNA in SD to a greater extent than WKY, as demonstrated by a strain × conditioning interaction [*F*(1,19) = 5.06, *p* < 0.05] (Figure 2). BDNF mRNA was increased by conditioning, main effect of conditioning [*F*(1,19) = 15.4, *p* < 0.001], and both strains showed learning-induced increases [SD: t(9) = 3.17, *p* < 0.05; WKY: t(10) = 2.4, *p* < 0.05]. In CA3, conditioning enhanced BDNF mRNA [*F*(1,20) = 12.94, *p* < 0.005] with a trend for upregulation in CA1 [*F*(1,19) = 3.57, *p* = 0.074], but these changes did not differ between strains.

#### TrkB Receptor

In all three subregions of the hippocampus, rats in the classical conditioning group had higher TrkB mRNA than sham rats [DG: *F*(1,19) = 8.09, *p* < 0.01; CA3: *F*(1,20) = 10.32, *p* < 0.005; CA1: *F*(1,20) = 6.24, *p* < 0.05] (Figure 2). However, TrkB mRNA did not differ between strains in any of the hippocampal subregions.

#### Arc

In the DG, classical conditioning upregulated Arc mRNA [*F*(1,19) = 4.67, *p* < 0.05] (Figure 2). Conditioning increased Arc mRNA to a greater extent in SD rats compared to WKY rats, main effect of strain [*F*(1,19) = 4.94, *p* < 0.05], strain × conditioning interaction [*F*(1,19) = 3.04, *p* = 0.097]. Arc mRNA did not differ between strains or conditioning groups in the CA1 and CA3 regions.

### Effects of intrahippocampal BDNF on delay eyeblink conditioning acquisition

**Figure 3:**
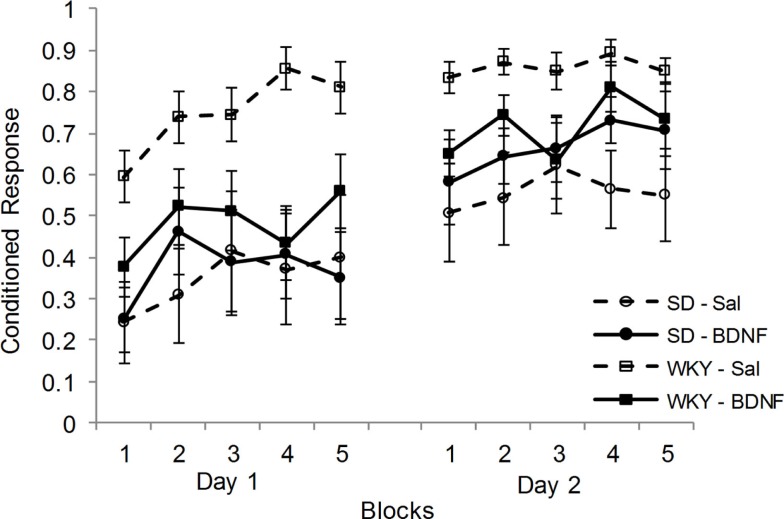
**Intrahippocampal BDNF normalized classical conditioning of the eyeblink response in the WKY rat**. BDNF was administered into the dentate gyrus of SD and WKY rats prior to each of two sessions of eyeblink conditioning. Saline-treated WKY rats acquired eyeblink conditioning significantly faster and to a greater extent than saline-treated SD rats, as demonstrated by more conditioned responses. BDNF administration in WKY rats slowed classical eyeblink conditioning to a level similar to that observed in SD rats. BDNF treatment did not alter classical conditioning in SD rats.

Following CCER, up-regulation of BDNF and Arc mRNA in the DG was blunted in the WKY rats compared to SD rats. Therefore, the effects of administering BDNF into the DG at the time of CCER were evaluated in both strains. Rats (SD-saline, *n* = 7; SD-BDNF, *n* = 8; WKY-saline, *n* = 9; WKY-BDNF, *n* = 9) were administered and conditioned in two sessions. Only animals that had reliable EMG signals on both days of training were used in the analysis. CRs increased as a consequence of training in all rats for days 1 and 2, main effect of block [*F*(4, 116) = 8.148, *p* < 0.001] and main effect of day [*F*(1, 29) = 24.28, *p* < 0.001] (Figure 3). Similar to previous studies, WKY rats acquired faster than SD rats, main effect of strain [*F*(1, 29) = 12.48, *p* < 0.001]. Importantly, BDNF infusion into the DG affected WKY but not SD rats, strain × treatment interaction [*F*(1, 29) = 4.972, *p* < 0.05]. Neither the main effect of treatment nor the block × strain × treatment interaction was significant.

Given the significant strain × treatment interaction, further analysis was conducted on the effects of BDNF in each strain. In SD rats, BDNF treatment did not alter the acquisition of delay CCER, as neither the main effect nor interactions involving treatment were significant. By contrast, WKY rats were significantly slowed in acquisition by BDNF administration, main effect of treatment [*F*(1, 16) = 8.7, *p* < 0.01] and treatment × day × block interaction [*F*(4, 64) = 2.72, *p* < 0.05].

## Discussion

The present study utilized the WKY rat to investigate the role of hippocampal BDNF in the facilitated associative learning that is observed in behaviorally inhibited individuals. The hippocampus was the focus of this study because it contains a high amount of BDNF (5–8), and dysfunction of hippocampus and BDNF systems both represent vulnerabilities for developing anxiety disorders (2, 9, 10, 20, 29). Furthermore, hippocampal damage leads to facilitated acquisition of delay CCER (39), similar to high behaviorally inhibited humans (41–44) and animals (45). In agreement with previous findings, the present study found WKY rats acquired delay CCER faster and to a greater degree than SD rats. Acquisition of CCER was associated with increased BDNF and Arc mRNA in the DG and CA3 of the hippocampus. Importantly, WKY rats had smaller increases than SD rats in the DG. TrkB mRNA was also increased following CCER in all hippocampal subregions, but these changes did not differ between strains. The smaller learning-induced changes of BDNF and Arc mRNA in WKY rats suggested that the lack of BDNF and resultant hippocampal dysfunction in this rat strain may be responsible for facilitated CCER. To test this hypothesis, exogenous BDNF was administered into the DG of SD and WKY rats prior to eyeblink conditioning sessions. Intrahippocampal BDNF slowed CCER acquisition of WKY rats to a level similar to SD rats. By contrast, BDNF infusions did not alter CCER acquisition in SD.

Brain-derived neurotrophic factor is important for hippocampal-dependent learning (61, 62). With respect to classical conditioning, contextual fear conditioning enhanced the number of CA1 neurons expressing BDNF immunoreactivity (63). BDNF heterozygous knockout mice were poorer in acquiring contextual but not cued fear conditioning, suggesting a differential action of BDNF on hippocampal-dependent and -independent forms of classical conditioning (64). In the present study, acquisition of a hippocampal-independent form of CCER increased BDNF mRNA in all three subregions of the hippocampus.

An increase in BDNF causes somatodendritic expression of Arc mRNA in the dentate gyrus (65). Arc, an immediate early gene, is one of the first genes transcribed after receiving extracellular signaling and is implicated in learning and memory. The induction of Arc enlarges dendrites, impacts dendritic structure and organization, is activated in dendrites in an NMDA-dependent manner (66), and is increased several hours post-BDNF infusion (67). Arc was increased in the hippocampus following hippocampal-dependent trace and contextual fear conditioning, but not after hippocampal-independent delay fear conditioning (68). The lack of change in Arc following hippocampal-independent delay fear conditioning contrasts with results of the present study, which showed increases in Arc mRNA following hippocampal-independent delay CCER. In the present study, the increase in Arc mRNA was only observed in the DG and not in the other hippocampal subregions. Therefore, the lack of change in Arc following fear conditioning may be due to dilution of the Arc changes in DG by other hippocampal subregions, although differences between fear conditioning and CCER cannot be entirely ruled out either.

Although WKY rats acquired delay CCER faster and to a greater extent than SD rats, they had smaller increases in BDNF and Arc mRNA than SD rats. Blunted changes in BDNF and Arc mRNA observed in WKY rats can be interpreted as poorer hippocampal function, and is supported by impaired hippocampal synaptic plasticity in WKY rats (56). Thus, our results support the view that damage or dysfunction of the hippocampus can lead to better acquisition of delay CCER (39).

Brain-derived neurotrophic factor administration enhances various forms of learning and memory (62). Infusion of BDNF into the hippocampus enhanced water maze reversal learning and reduced anxiety-like behavior in an elevated plus maze, suggesting that hippocampal BDNF improve hippocampal-dependent learning and reduce anxiety (69). Additionally, hippocampal infusions of BDNF enhanced contextual fear conditioning in BDNF heterozygous knockout mice (64) and transgenic mice expressing active CREB or their wild-type counterparts (70). While most evidence is that BDNF enhances hippocampal-dependent forms of learning, the effect of hippocampal BDNF administration on hippocampal-independent learning has not been addressed. The present study shows that administration of BDNF into the hippocampus of WKY rats slowed acquisition of delay CCER to a level equivalent to that demonstrated by SD rats. Thus, hippocampal BDNF administration can result in poorer acquisition on some forms of learning and in some rat strains. In this regard, the delay CCER paradigm may be a special case because hippocampal damage can facilitate acquisition (39).

The results of the present study provide a potential link between three anxiety vulnerabilities: BDNF dysfunction, small hippocampal volume and impaired function, and behavioral inhibition. BDNF dysfunction can lead to reduced hippocampal volume and impaired hippocampal-dependent learning. In humans, abnormally low levels of BDNF are associated with a smaller hippocampal volume (22). However, the effect of the BDNF Val66Met SNP on hippocampal volume in humans is unclear (71), although an association between reduced hippocampal volume and the interaction of Val66Met SNP with environmental factors (childhood maltreatment) is growing (72, 73). Individuals with the Val66Met SNP have impairments in learning and memory that are generally considered to be hippocampal dependent (74). Mice with the Val66Met SNP have smaller hippocampi, reduced activity-dependent secretion of BDNF, dendritic shrinkage in the DG, and impaired extinction of fear conditioning compared to wild-type mice (9, 29). It is possible that BDNF and hippocampal dysfunction represent the same vulnerability. Early childhood trauma or chronic stress is a risk factor for anxiety disorders. One of the structures most affected by chronic stress is the hippocampus, due to the density of glucocorticoid receptors (GRs) and its involvement in regulating the HPA axis (75–77). One mechanism by which stress has a negative impact on hippocampal morphology and function is by decreasing hippocampal BDNF, resulting in decreased neurogenesis, dendritic atrophy, and impaired cognition (3, 4, 28, 78–80). These stress-induced reductions of BDNF may relate to the reductions of BDNF protein and hippocampal volume in some patients without Val66Met genotype.

While there is an abundance of evidence associating BDNF and hippocampus volume and function, links between inhibited temperament and BDNF or hippocampal dysfunction has been sparse. Individuals with inhibited temperament have abnormal hippocampal processing of novel stimuli in humans (37, 81). Interestingly, activation of the hippocampus to novel faces was most strongly associated with inhibited temperament *and* childhood maltreatment (37). As described above, childhood maltreatment and chronic stress are associated with smaller hippocampal volume and hippocampal dysfunction. In animal studies, the behaviorally inhibited WKY rat has a smaller hippocampus than non-inhibited rat strains (56), impaired hippocampal synaptic plasticity (56), and poorer performance on hippocampal-dependent learning procedures (49, 57). The WKY rat also behaves similarly to rats with hippocampal damage (56, 58). Thus, there is little evidence to link inhibited temperament with smaller hippocampus or BDNF dysfunction, except for the animal work. However, inhibited temperament may interact with either BDNF/hippocampal dysfunction to exacerbate vulnerability to develop anxiety disorders.

In summary, BDNF dysfunction in the hippocampus was observed in an animal model of behavioral inhibition, the WKY rat. This dysfunction was related to facilitated acquisition of hippocampal-independent associative learning. Gain of function experiments by administering BDNF into the hippocampus of WKY rats “normalized” associative learning. The results suggest a possible mechanism by which hippocampal dysfunction and behavioral inhibition leads to pathological associative learning and vulnerability to develop anxiety disorders.

## Conflict of Interest Statement

The authors declare that the research was conducted in the absence of any commercial or financial relationships that could be construed as a potential conflict of interest.
